# Predicting prognosis and immune status in sarcomas by identifying necroptosis-related lncRNAs

**DOI:** 10.18632/aging.205383

**Published:** 2024-01-08

**Authors:** Zhen Wang, Anfang He, Zhengyu Lu, Wenli Xu, Gang Wu, Tingsheng Peng

**Affiliations:** 1 Department of Pathology, The First Affiliated Hospital of Sun Yat-Sen University, Guangzhou 510080, Guangdong, China

**Keywords:** necroptosis, sarcomas, risk model, SNHG6, TME

## Abstract

Background: Sarcomas are a type of highly heterogeneous malignant tumors originating from mesenchymal tissues. Necroptosis is intricately connected to the oncogenesis and progression of tumors. The main goal of this research is to assess the prognostic value of necroptosis-related lncRNAs (NRlncRNAs) in sarcomas and to develop a risk model based on NRlncRNAs to evaluate prognostic and immune status of the sarcomas.

Methods: We screened NRlncRNAs using the gene co-expression network, developed a prognostic risk model of sarcomas, and then verified the model. Following that, various bioinformatics analysis algorithms were employed to analyze the distinct characteristics of patients of the risk model. Furthermore, the function and regulatory mechanism of NRlncRNA SNHG6 in sarcomas were investigated through osteosarcoma cell experiments, such as qRT-PCR, Western blot, CCK-8, clone formation, and transwell assay.

Results: We successfully developed a NRlncRNAs-related prognostic risk model and screened 5 prognosis-related NRlncRNAs, with SNGH6 being the most significant for prognosis of patients. According to results, the significant differences exist in prognosis, clinical characteristics, and tumor immune status among patients of the risk model. The experiments of osteosarcoma cells demonstrated that NRlncRNA SNHG6 knockdown significantly attenuated the cells’ proliferation, migration, and invasion. qRT-PCR and WB results showed that SNHG6 regulated AXL and AKT signaling.

Conclusions: We have developed an innovative investigation on NRlncRNAs, which can serve as a reference for diagnosis, therapy, and prognosis of sarcomas. Additionally, we demonstrated that NRlncRNA SNHG6 regulated AXL and AKT signaling in osteosarcoma cells and the proliferation, migration, and invasion of tumor cells.

## INTRODUCTION

Sarcomas, originating from mesenchymal tissue, are highly heterogeneous malignant tumors consisting of over 170 subtypes [1]. Statistics show that a total of 34,270 new sarcoma-related cases and 15,086 sarcoma-related deaths were identified worldwide in 2020 [2]. Sarcomas, which primarily arise from soft tissue (approximately 87%) and bone tissue (approximately 13%), constitute around 1% of malignant tumors in adults and 15% of malignant tumors in children [1, 3]. The survival rate for sarcomas in adults varies between 53% and 60% [4].

At present, the standard therapeutic schedule for sarcoma patients is surgery with adjuvant chemotherapy or radiotherapy. Despite continuous optimization of therapeutic schedule in recent years, 35%-45% of sarcoma patients developed tumor metastasis and had a poor prognosis [3]. Due to the tumor compensatory mechanism, tumor heterogeneity and complex tumor microenvironment (TME), targeted therapy for sarcomas is limited to partial tumor subtypes [5]. As tumor research has progressed, TME has shown a notable impact on the progression of sarcomas, and immunotherapy has become a novel therapeutic choice for sarcomas [6, 7]. Therefore, it is particularly important to identify new biomarkers and understand their importance in TME for finding new potential therapeutic strategies of sarcomas and improving the prognosis of sarcoma patients [8].

Necroptosis, a kind of regulatory cell death, has well-defined effector mechanisms and can be regulated by multiple signaling pathways [9, 10]. The regulation of necroptosis primarily relies on the phosphorylation of mixed-lineage kinase domain-like protein (MLKL) by receptor-interacting protein kinase-1 (RIPK1) and RIPK3 [11]. When phosphorylated RIPK1 and RIPK3 form a high molecular weight complex, MLKL is recruited and phosphorylated to form a necrosome complex. Subsequently, the necrosome complex induces cell membrane lysis and cell necrosis, releasing pro-inflammatory signals and activating the immune system [12, 13]. On the one hand, necroptosis can trigger adaptive immunity, which consequently hinders the progression of tumors. For example, necroptotic cells introduced ectopically into melanoma TME may enhance their anti-tumor immunity [14]. On the other hand, necroptosis can in turn produce immune suppressive TME, which promotes tumor growth. Necroptotic cells in pancreatic cancer can induce the release of chemokine attractant CXCL1 and the transduction of Mincle signaling, causing macrophage-induced immune suppression, forming an immunosuppressive TME, and promoting migration and invasion of cancer cell [15]. Thus, necroptosis appears to have complex context-dependent tumor suppressive or promotive effects. Furthermore, despite the extensive investigation into the molecular mechanisms of necroptosis, there remains a lack of complete understanding regarding the precise control and role of necroptosis in the development and progression of sarcomas. Therefore, it is imperative to thoroughly investigate the influence of necroptosis on sarcomas.

LncRNAs can be widely found in all types of tumors. Despite their inability to code proteins, lncRNAs contribute to the regulation of various tumor genes [16]. According to studies, lncRNAs have been found to be linked with necroptosis. For example, lncRNA PVT1 promotes hepatocellular necroptosis by increasing ZBP1 promoter methylation [17], and lncRNA 107053293 regulates necroptosis in chicken tracheal cells by antagonizing miR-148a-3p [18]. Furthermore, several evidences suggest that lncRNA may be important in the diagnosis and prognosis of tumors and may serve as a new target for future cancer treatment [19, 20]. Nonetheless, the predictive value and potential function of lncRNA on necroptosis of sarcoma cells are still incompletely well known. Therefore, it is imperative to thoroughly examine the association between NRlncRNAs and the sarcoma prognosis, along with their connection to the tumor immune microenvironment.

Through an extensive bioinformatics analysis that combines genomics and clinical data, we have created a risk model of sarcomas using NRlncRNAs. Potential values of the model, in terms of clinical characteristics, differentially expressed genes, pathways and immune cell infiltration, were also explored. Then, we validated the NRlncRNA SNHG6 in osteosarcoma cells. Overall, the diagnosis and treatment of sarcomas may be enlightened by this study.

## MATERIALS AND METHODS

### Data collection

The data of TCGA-SARC were downloaded from UCSC Xena [21]. A grand total of 256 samples were collected, comprising of 158 samples that were alive and 98 samples that were deceased. 601, 159, 8 and 45 necroptosis-related mRNAs were obtained respectively by searching the keyword “necroptosis” in GeneCards, Kyoto Encyclopedia of Genes and Genomes (KEGG), Molecular Signatures Database (MSigDB) and Gene Ontology (GO) (Supplementary Table 1) [22–25]. Next, we downloaded the GEO datasets: GSE39057 [26], GSE39055 [26], GSE17674 [27] along with the clinical data. These datasets were merged to create a separate external dataset for the purpose of validating the risk model. This external dataset consisted of 48 alive samples and 38 dead samples. The data of BOCA-FR were downloaded from the ICGC [28], while the data of TARGET-OS were downloaded from the UCSC Xena. The aforementioned two datasets were merged to create another separate external dataset, comprising of 86 alive samples and 56 dead samples.

### Identification of NRlncRNAs

The extracted 712 necrosis-related mRNAs were paired with all the lncRNA expression profile of TCGA-SARC by Pearson correlation test to obtain significantly related mRNA-lncRNA pairs. Cytoscape software [29] was adopted to develop a necroptosis-related co-expression network, in which the lncRNA was NRlncRNAs. Additionally, the R package “ggplot2” and “pheatmap” were utilized to draw the mRNA-lncRNA correlation heatmap and the NRlncRNA expression heatmap.

### Construction of a prognostic predictive risk model

TCGA-SARC clinical characteristics were analyzed by LASSO-COX to identify prognosis-related risk genes. The best lambda value was selected, and only the genes whose coefficients are not zero were retained as prognosis-related lncRNAs after regression. The risk model was constructed by selecting risk coefficients corresponding to prognosis-related lncRNAs. Then, the risk scores of patients were calculated, and the R package “survival” was utilized to assess the ideal grouping threshold of the risk scores of patients. The KM survival analysis was conducted on patients in high- and low-risk groups using the R package “survminer”, which enabled the calculation of prognosis time the difference between the two groups. The risk model’s efficiency was assessed by utilizing the R package “timeROC”. R package “ggplot2” and “pheatmap” were utilized to draw the triple plot of prognostic risk based on clinical information of patients. Subsequently, based on the grouping threshold of the training set, we grouped two external independent datasets to verify the accuracy of the risk model.

### GSEA and functional annotation

The R package “org.Hs.eg.db” and “clusterprofiler” were employed to perform GO functional annotation and KEGG pathway enrichment for the mRNA that showed significant association with the NRlncRNAs in the co-expression network. Then, GSEA was conducted in the risk model of NRlncRNAs.

### Analysis of differences in clinical characteristics

The R package “survival” was utilized to perform univariate and multifactor Cox regression analyses on the risk score and clinical characteristics of patients in TCGA-SARC data. Additionally, the R package “forestplot” was employed to create a forest plot illustrating the outcomes. The R packages “rms” and “Hmisc” were utilized to construct the nomogram and calibration curve. R package “pheatmap” and “ggpubr” was utilized to draw the heatmap and box plot between risk genes and clinical characteristics.

### Analyses of immune status and drug susceptibility in the risk model

To analyze immune status and drug susceptibility in the risk model, R package “ggpubr” was used to draw immune checkpoint box plot, and “ESTIMATE”, “ssGSEA”, “CIBERSORT” and “oncoPredict” were adopted to evaluate immune cell infiltration and drug susceptibility, respectively.

### Transfection and culture of cells

In a humidified incubator with 5% CO_2_ at 37° C, osteosarcoma cells 143B and MG63 cells were grown in DMEM (Gibco, NY, USA) with 10% fetal bovine serum (Gibco, NY, USA) and 1% penicillin-streptomycin solution. RiboFECT™ CP Transfection Kit (RiboBio, Guangzhou, China) was used to transfect 143B and MG63 cells with siRNAs (RiboBio, Guangzhou, China). Then, transfected 143B and MG63 cells were incubated for 48 hours to isolate RNA and proteins. The target sequences of siRNA were documented in Supplementary Table 2.

### Analyses of qRT-PCR and Western blot (WB)

RNA was isolated utilizing Trizol reagent (Invitrogen, CA, USA), and cDNA was synthesized using the PrimeScript RT reagent Kit (Takara, Nanjing, China). The SYBR Premix Ex Taq II (Takara, Nanjing, China) was utilized for qRT-PCR, and the 2-ΔΔCt method was employed for relative quantification. The sequences of primers were documented in Supplementary Table 2. Proteins were isolated from transfected 143B and MG63 cells using RIPA lysis solution (P0013B, Beyotime, China) and a mixture of protease and phosphatase inhibitors (P1045, Beyotime, China). Protein samples were separated using SDS-PAGE gels (P0012AC, Beyotime, China) and PVDF membrane (Merck Millipore, MA, USA) was utilized to transfer protein. The membrane was obstructed in a solution containing 5% BSA at room temperature for 1 hours. After overnight usage of the primary antibody incubation membrane at 4° C, HRP-conjugated anti-rabbit secondary antibody (1:5000; 7074P2; CST, USA) was used to incubate the membrane at room temperature for 1 hour. The antibodies were used: AXL (1:1,000; 8661S; CST), phospho-AXL (1:1,000; 5724S; CST), AKT (1:1,000; 4691S; CST), phospho-AKT (1:2,000; 4060S; CST), and GAPDH (1:1,000; 2118S; CST).

### CCK-8, clone formation, migration and invasion assays

Osteosarcoma cells transfected with siRNA were inoculated into 96 well plates at a density of 1000 cells per well. At the specified time intervals, 10ul of Cell Counting Kit-8 (KGA317, KeyGEN BioTECH, China) were introduced. Following the 2-hour incubation at 37° C in a cell incubator containing 5% CO_2_, the absorbance at 450nm was determined using the multifunctional enzyme marker. The osteosarcoma cells that were transfected were inoculated in a 6-well plate with 500 cells per well and then incubated 2 weeks. Next, the cell colonies were treated with 0.4% paraformaldehyde for fixation and then stained using 0.2% crystalline violet. Subsequently, the number of colonies with more than 50 cells was counted. Serum-free DNEM was used to prepare suspensions of osteosarcoma cells that were transfected. The suspension containing 5×10^4^ cells were inoculated into transwell chambers (Corning, NY, USA) precoated or uncoated with Matrigel solution. Then, 600 ul complete culture medium containing 20% FBS were added into the lower chamber. The cells were cultured for 48 hours. After that, the chambers were treated with 0.4% paraformaldehyde and subsequently stained using 0.2% crystalline violet. Cells that had invaded or migrated into the lower chamber were counted and imaged.

### Statistical analysis

R (version 4.3.0) was utilized for data calculations and statistical analyses. The independent t-test was utilized to estimate the statistical significance of variables following a normal distribution, while the differences between variables with an abnormal distribution were analyzed utilizing the Wilcoxon rank sum test. Statistical significance was determined by considering *P* < 0.05.

### Data availability

The public datasets analyzed in this study can be found at https://ocg.cancer.gov/ and https://www.ncbi.nlm.nih.gov/gds/.

## RESULTS

**Figure 1:**
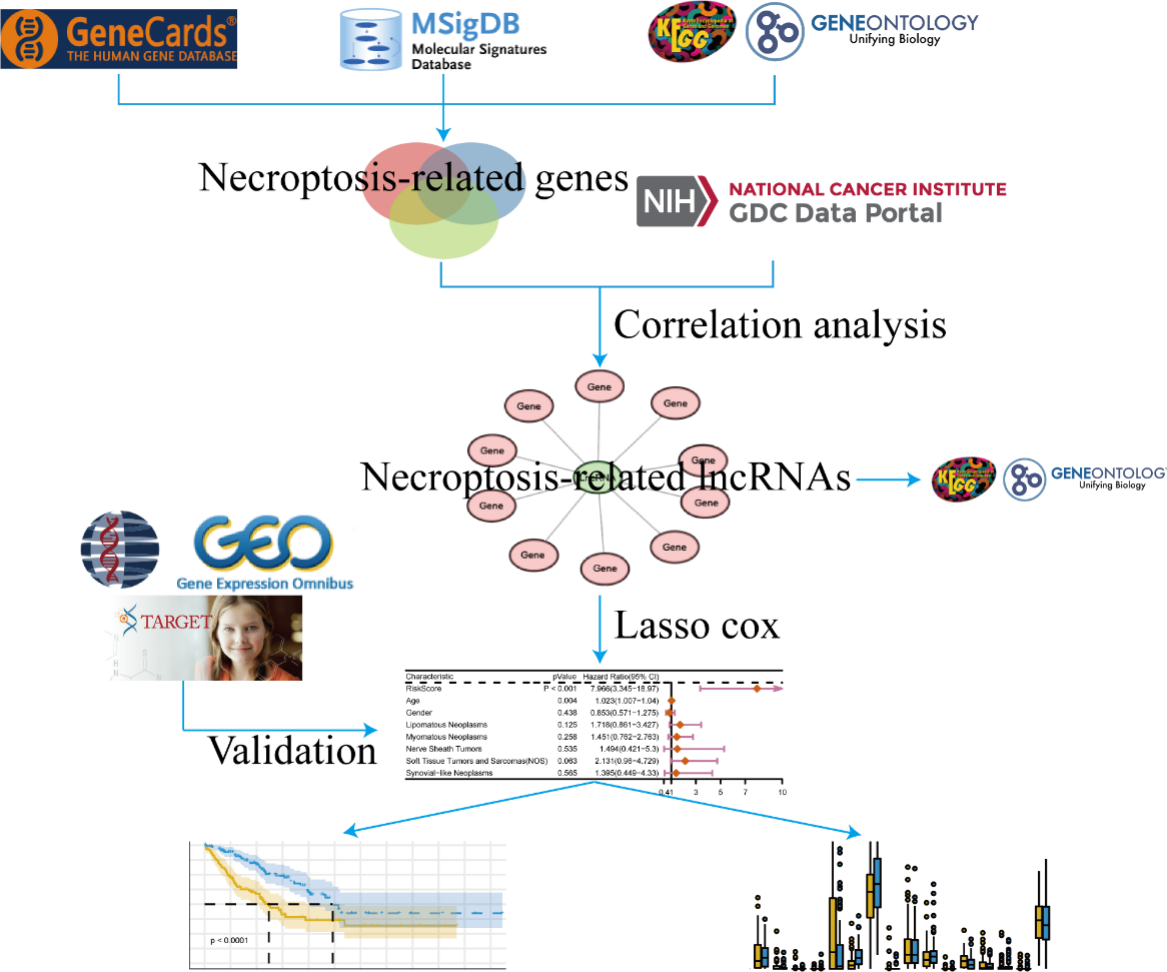
Flow chart.

Figure 1 shows the flow chart of this study.

### Identification of NRlncRNAs

**Figure 2:**
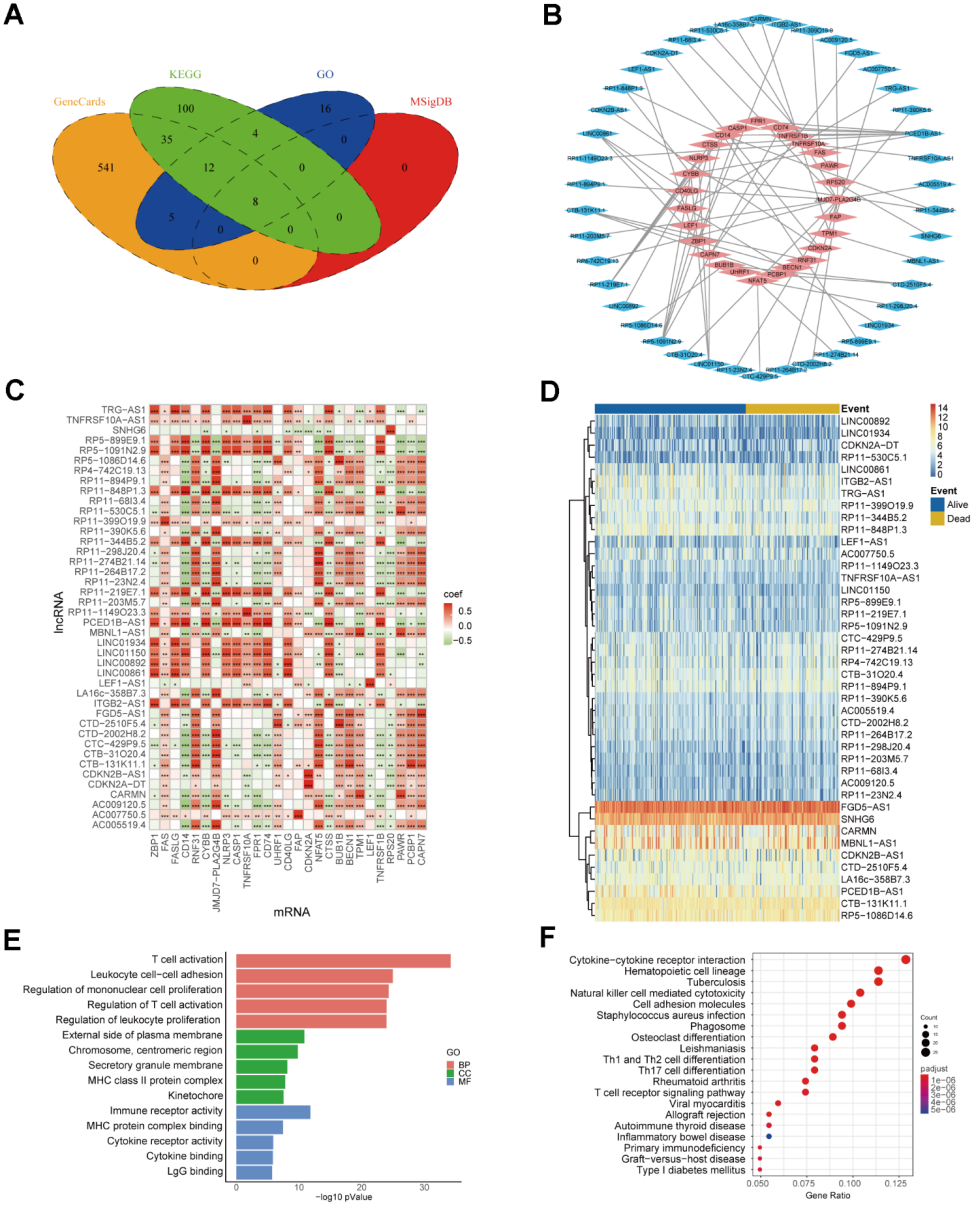
**Identification of NRlncRNAs.** (**A**) Necroptosis-related mRNA from Venn diagram. (**B**) Co-expression network of necroptosis-related mRNA-lncRNA. The blue part represents 42 lncRNAs, and the red part represents 27 mRNAs. (**C**) Heatmap of necroptosis-related mRNA-lncRNA correlation (* *P ≤* 0.05, ** *P ≤* 0.01, *** *P ≤* 0.001). (**D**) Heatmap of NRlncRNA expression. (**E**) The top 5 results for GO enrichment of necroptosis-related mRNAs. (**F**) The top 20 results for KEGG enrichment of necroptosis-related mRNAs.

Co-expression network of genes serves as a valuable approach for functional gene annotation and identification of unknown functional genes by linking them with biological processes. Hence, constructing necroptosis-related mRNA-lncRNA co-expression network can facilitate the identification of NRlncRNAs. At first, we searched in the GeneCards, MSigDB, KEGG and GO databases to obtain 721 necroptosis-related mRNAs (Figure 2A). Then, 64 mRNA-lncRNA co-expression pairs were obtained after 721 necroptosis-related mRNAs were interacted with all the lncRNAs of TCGA-SARC data (Figure 2B, 2C). LncRNAs in the co-expression network were highly correlated with necroptosis-related mRNAs, namely, 42 NRlncRNAs were screened by mRNA-lncRNA co-expression network. The heatmap of NRlncRNA expression also showed the differential trend in lncRNA expression (Figure 2D). We conducted GO and KEGG enrichment analysis by extracting 27 necroptosis-related mRNAs from the co-expression network, aiming to further expose the biological differences and describe the biological roles of NRlncRNAs in sarcomas (Figure 2E, 2F and Supplementary Table 3). The 5 most important terms from the GO and KEGG enrichment analysis were all associated with immune processes, indicating that sarcomas NRlncRNAs may have biological functions related to immune regulation.

### Construction of NRlncRNAs-related prognostic risk model

**Figure 3:**
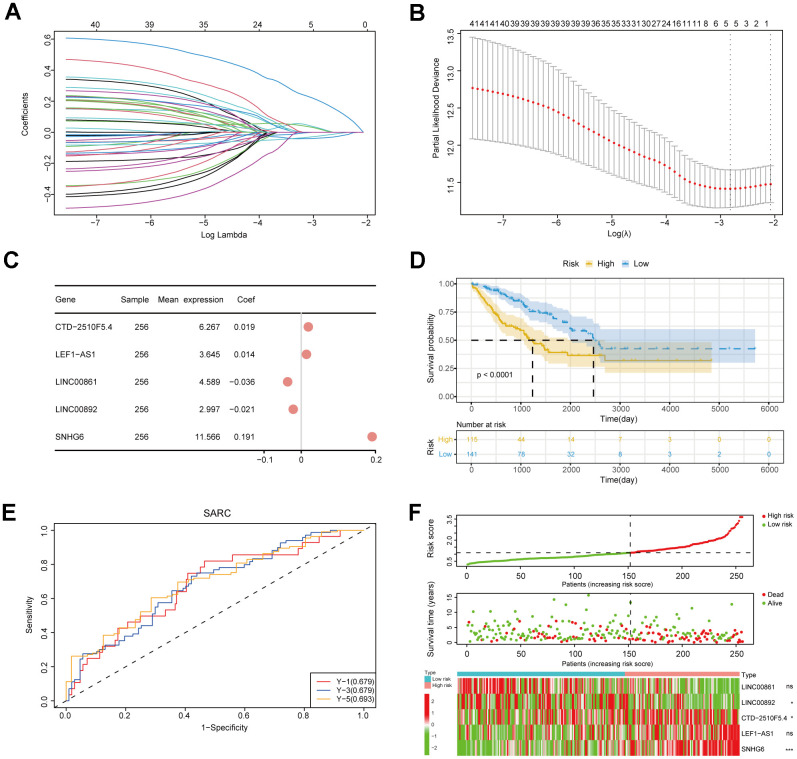
**Construction of the risk model.** (**A**) Lasso regression curve. This plot illustrated the screening of the Lasso regression for the 42 gene features. (**B**) Lambda value selection curve. The best lambda values of regression model were selected using this plot. (**C**) An analysis of prognostic risk genes by the forest plot. (**D**) The survival curve of patients. (**E**) ROC curves for the risk model. (**F**) The triple risk plot. The first diagram was the predicted risk values for each patient. The second diagram showed the relationship between the patients ranked by the predicted risk values and the survival status. The third diagram illustrated the relationship between lncRNA expressions (* *P* ≤ 0.05; ** *P* ≤ 0.01; *** *P* ≤ 0.001; ns, no significance).

**Figure 4:**
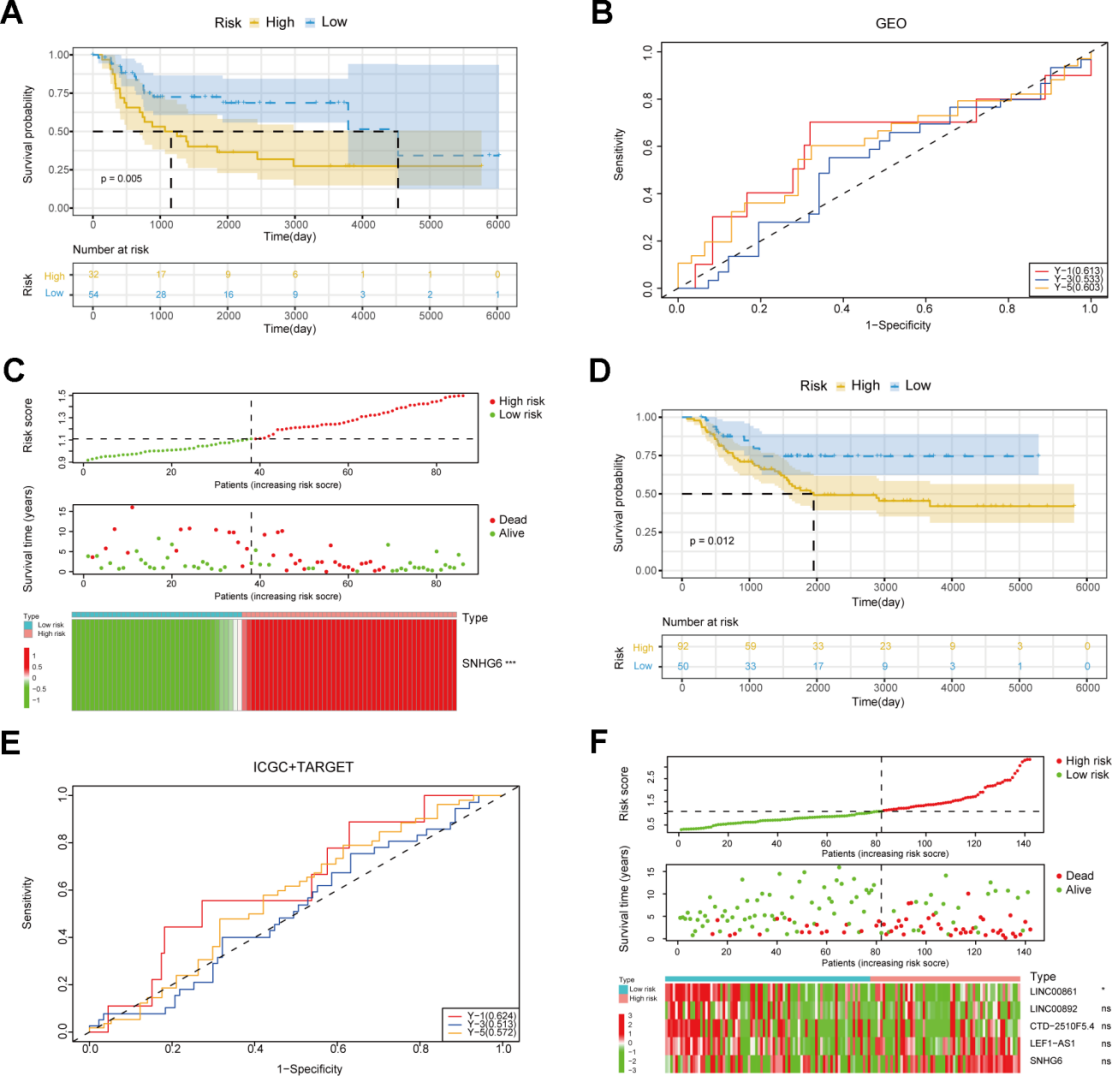
**Verification of the risk model.** (**A**) Survival curves of patients in the GEO cohort. (**B**) ROC curves in the GEO cohort. (**C**) The triple risk plot in the GEO cohort (* *P* ≤ 0.05; ** *P* ≤ 0.01; *** *P* ≤ 0.001; ns, no significance). (**D**) Survival curves of patients in the ICGC and TARGET cohorts. (**E**) ROC curves in the ICGC and TARGET cohorts. (**F**) The triple risk plot in the ICGC and TARGET cohorts (* *P* ≤ 0.05; ** *P* ≤ 0.01; *** *P* ≤ 0.001; ns, no significance).

In order to further evaluate NRlncRNAs that are related to prognosis, we developed a predictive model for the prognosis of NRlncRNAs using 42 selected NRlncRNAs and survival data of patients. Initially, the ideal lambda value was calculated using LASSO-COX (Figure 3A, 3B), followed by the retention of 5 lncRNAs associated with prognosis (Figure 3C). Then, we extracted risk coefficients associated with the 5 prognosis-related lncRNAs, which were used to calculate the risk score and develop a risk model. Risk score = -0.0365 * LINC00861 + -0.0214 * LINC00892 + 0.0191 * CTD-2510F5.4 + 0.0142 * LEF1-AS1 + 0.1909 * SNHG6. By utilizing the surv_cutpoint function, we identified the most suitable threshold for patients’ risk scores, subsequently categorizing them into groups of high and low risk (cut-off value = 1.109737). The patients were compared using KM analysis to assess overall survival (OS), revealing a significant difference in the result (*P* < 0.0001) (Figure 3D). We graphed ROC curves to confirm the model’s predictive ability. The result showed that the risk model is reliable, as indicated by all the AUC values being above 0.5 (Figure 3E). In addition, the triple risk plot indicated that among the 5 prognosis-related lncRNAs, the low-risk group exhibited a tendency towards high expression of LINC00861and LINC00892 whereas the high-risk group showed a tendency towards high expression of CTD-2510F5.4, LEF1-AS1 and SNHG6 (Figure 3F). Afterwards, we verified the risk model in two external cohorts to assess the accuracy of the model. The first cohort integrated three datasets of GSE39057, GSE39055 and GSE17674. In the first cohort, solely SNHG6 in the risk model was incorporated, and patients were divided into high- and low-risk groups based on risk scores. The two groups were validated for difference in OS using KM analysis, which revealed a significant difference (*P* = 0.005) (Figure 4A). The results from the ROC curves indicated that the model had good predictive ability, as evidenced by all the AUC values being above 0.5 (Figure 4B). The triple risk plot indicated that SNHG6 exhibited a tendency towards elevated expression in the high-risk group (Figure 4C). The second cohort integrated BOCA-FR and TARGET-OS. The KM analysis revealed a noteworthy difference in OS between the two groups (*P* = 0.012). All the AUC values for the model were above 0.5 (Figure 4E). Based on the triple risk plot, similarly, SNHG6 exhibited a tendency towards high expression in the high-risk group (Figure 4F).

### Prognostic analysis of sarcoma patients

**Figure 5:**
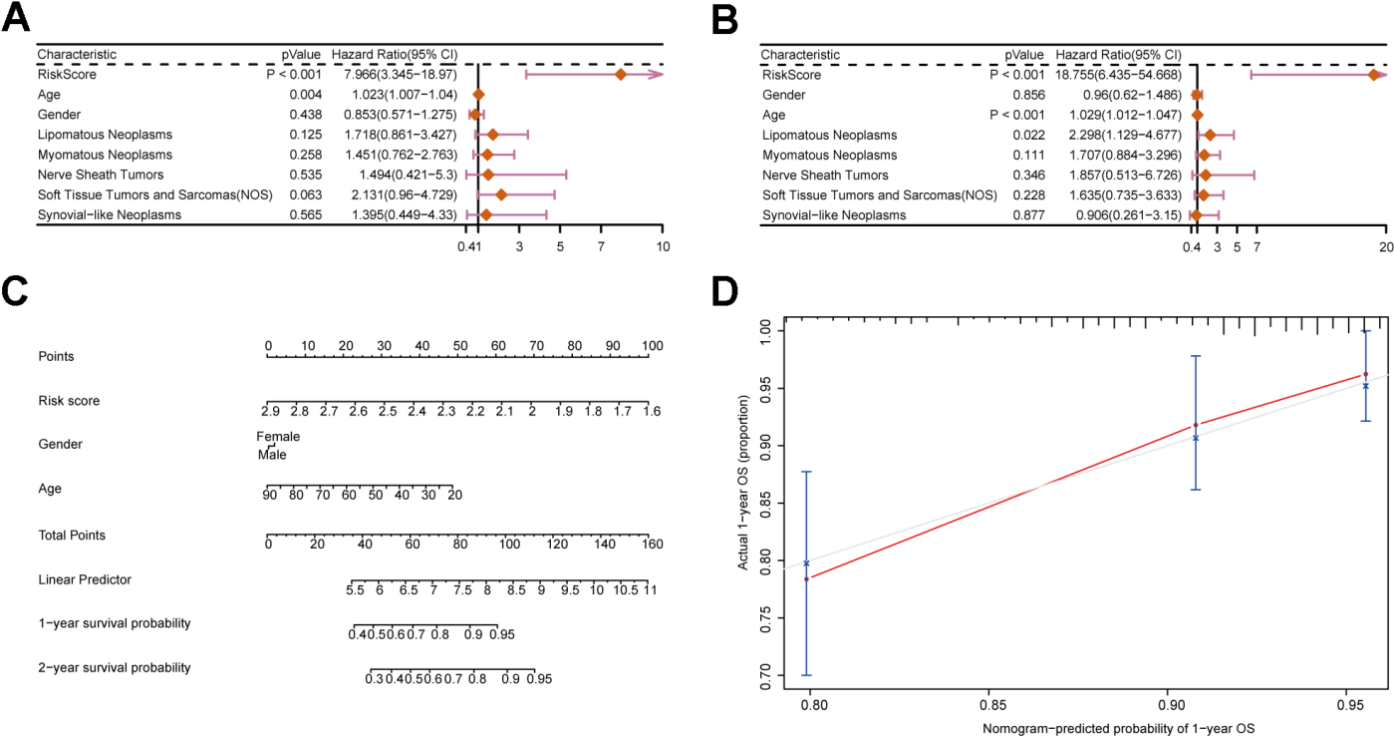
**Construction of a nomogram incorporating clinical characteristics.** (**A**, **B**) Forest plots displaying clinical characteristics and risk scores. (**C**) Nomogram of sarcoma patients. (**D**) Calibration curve for the nomogram.

To assess the influence of clinical characteristics on the prognosis of sarcoma patients and establish whether the risk score acts as an independent risk factor, we conducted univariate and multivariate Cox regression analyses on various clinical characteristics and risk score (Supplementary Table 4). The forest plot demonstrated the risk score as independence risk factor for patients’ prognosis (Figure 5A, 5B). Next, we utilized a nomogram to visualize the influence of each clinical characteristics on the outcome variable. The outcome revealed that the risk model had the greatest predictive capacity for both 1- and 2-year survival (Figure 5C). The nomogram result was further supported by the calibration curve (Figure 5D). To sum up, our prognostic model’s score is an excellent predictor of sarcoma patients’ prognosis, and it is independent of multiple clinical characteristics.

### Correlation analysis between clinical characteristics and risk score

**Figure 6:**
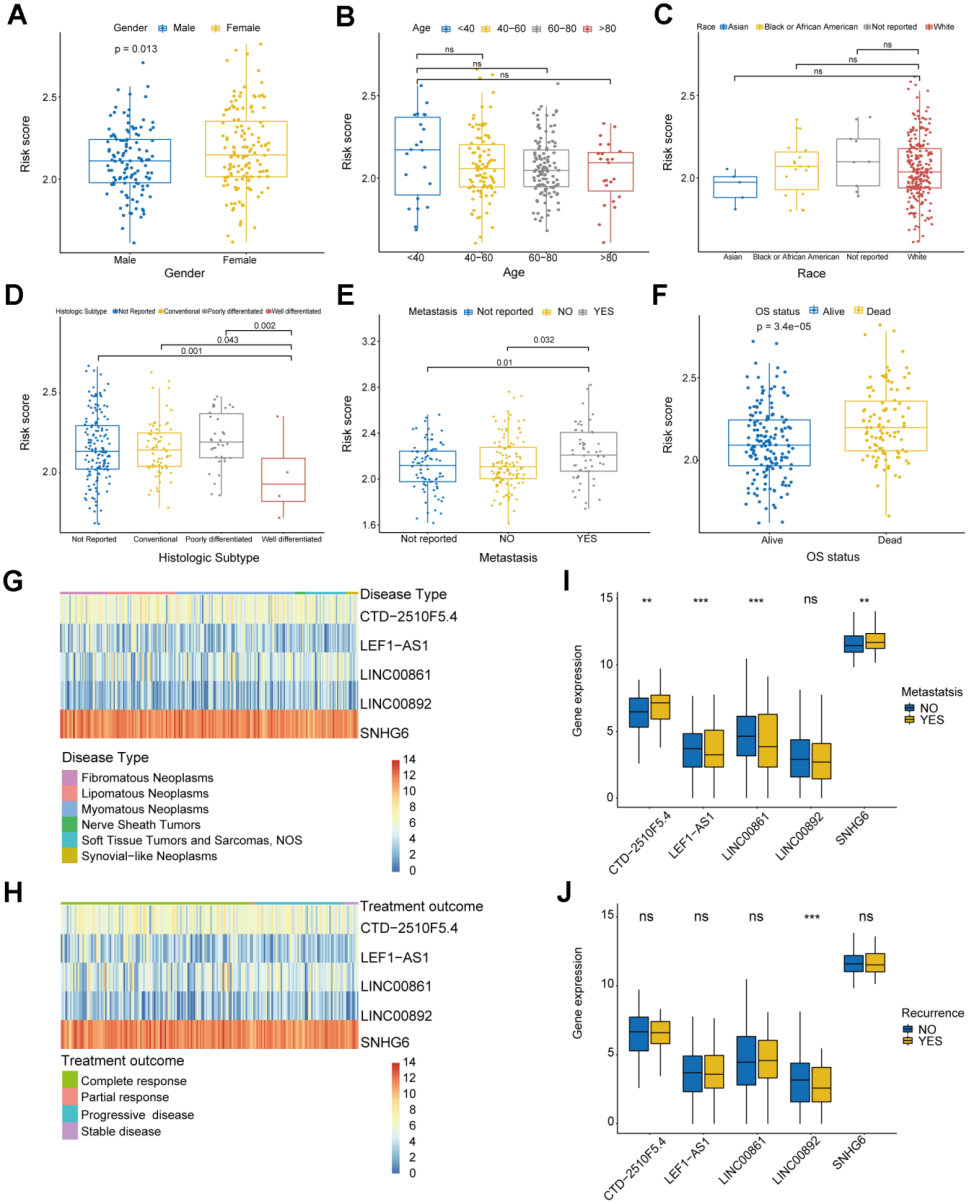
**Analysis of clinical characteristics in risk model.** (**A**) Box plot displaying the gender group and risk score. (**B**) Box plot displaying the age group and risk score. (**C**) Box plot displaying the race group and risk score. (**D**) Box plot displaying the histologic subtype group and risk score. (**E**) Box plot displaying the metastatic status group and risk score. (**F**) Box plot displaying the OS status group and risk score. (**G**, **H**) Heatmaps of correlation between risk genes and disease type and treatment outcome. (**I**, **J**) Box plots of correlation between risk genes and metastatic status and recurrence. (* *P* ≤ 0.05; ** *P* ≤ 0.01; *** *P* ≤ 0.001; ns, no significance).

In order to investigate clinical characteristics in risk model, we selected clinical information of patients, including gender, age, race, histologic subtype, metastatic status, OS status, disease type, recurrence, and treatment outcome, to draw baseline data chart (Supplementary Table 5). The result showed that the gender group revealed significant difference between males and females (Figure 6A). In the age group, it was evident that the risk score of patients below 40 years old was considerably greater compared to the other groups, yet the difference in risk score did not show statistical significance (Figure 6B). Figure 6C shows that there were no significant differences in the race group, suggesting that the clinical prognosis of sarcomas may not be influenced by race. In histologic subtype, significant differences were shown in risk score of well differentiated sarcomas and conventional and poorly differentiated sarcomas (Figure 6D). Moreover, significant differences existed between the groups with metastasis and without metastasis, and the metastatic group exhibited a higher risk score, indicating that the risk model has the capability to forecast sarcoma metastasis (Figure 6E). Significant difference in risk score was also observed among the OS status group (Figure 6F). To further explore the specificity of risk genes among different clinical characteristics, we analyzed 4 clinical characteristics: disease type, treatment outcome, metastatic status and recurrence. The visualized heatmaps showed that SNHG6 had the highest expression level among 5 risk genes of sarcoma patients with any disease types and treatment outcomes, whereas LNC00892 exhibited the lowest expression level (Figure 6G, 6H). In the metastatic group, SNHG6 and CTD-2510F5.4 exhibited elevated expression levels compared to the non-metastatic group, whereas LEF1-AS1, LINC00861, and LINC00892 showed the opposite trend (Figure 6I). LINC00892 of recurrent patients showed a trend of low expression compared to non-recurrent patients (Figure 6J). All of these results reveal that the risk genes show different expression trends in sarcoma patients with different clinical characteristics and prognostic outcomes, which demonstrates the validity of screened risk genes.

### Analysis of immune status of the risk model

**Figure 7:**
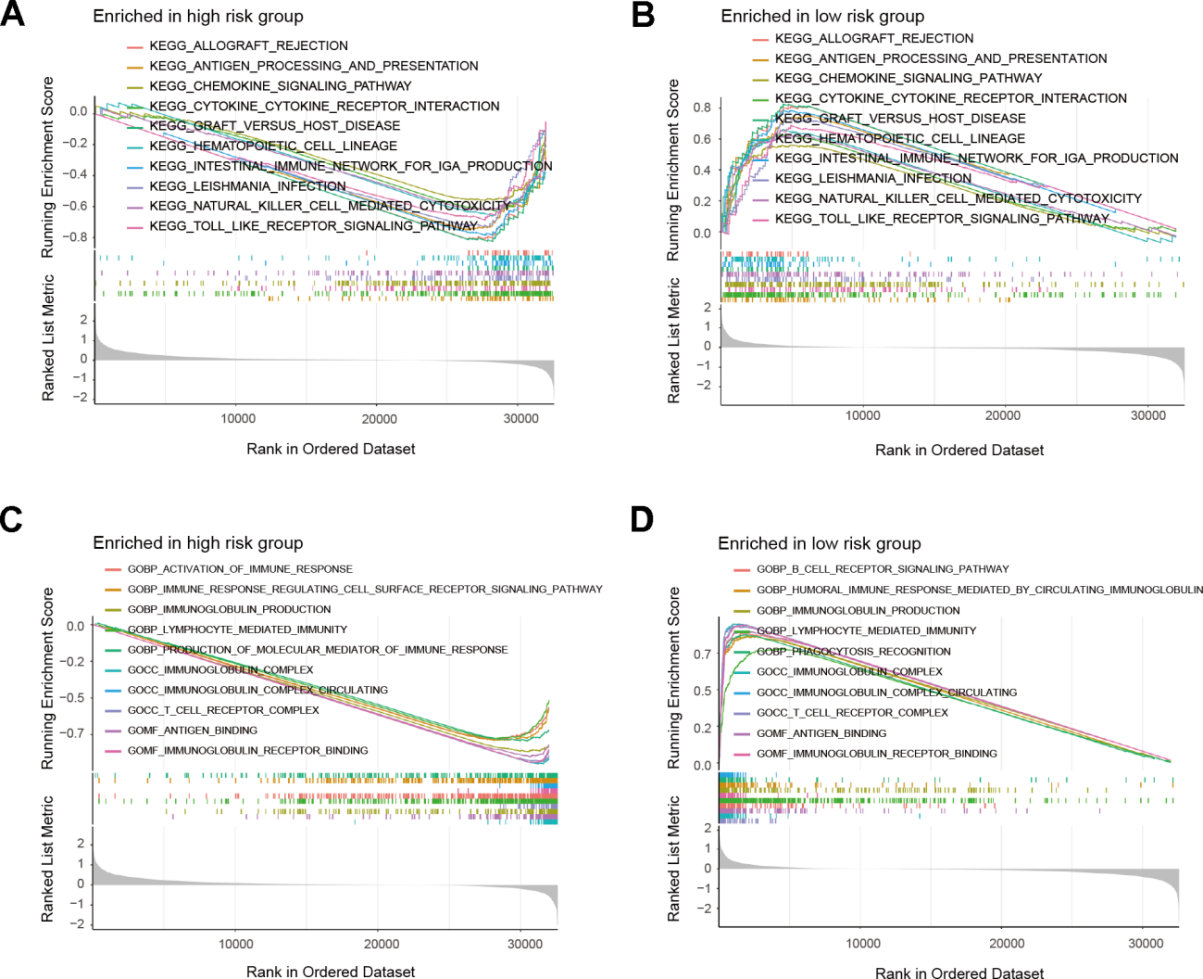
**GSEA results of risk model.** (**A**, **B**) Top 10 results for KEGG enrichment of patients in the risk model. (**C**, **D**) Top 10 results for GO enrichment of patients in the risk model.

**Figure 8:**
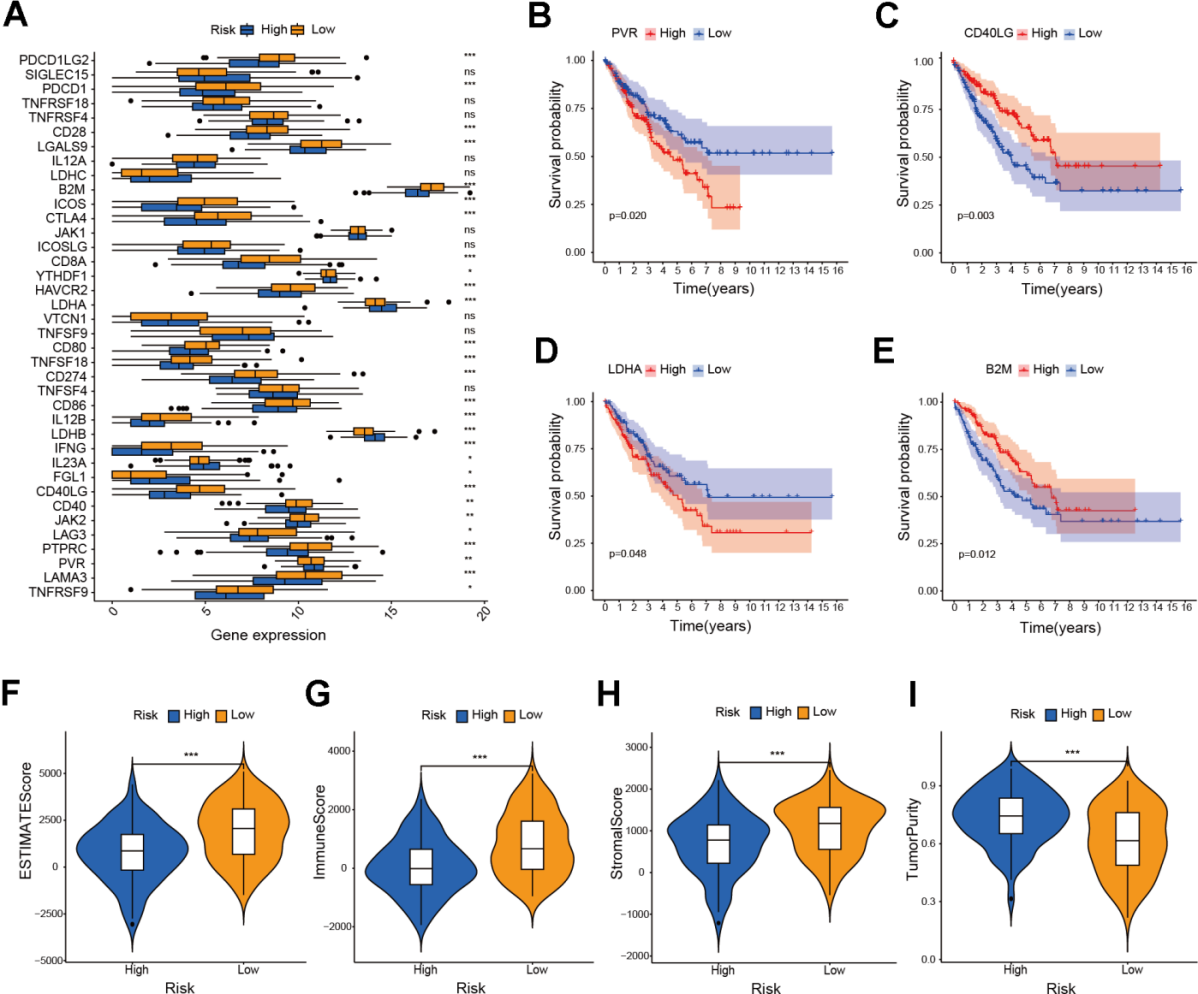
**Investigation of ICPs and ESTIMATE score in risk model.** (**A**) Analysis of immune checkpoint genes in the risk model (* *P* ≤ 0.05; ** *P* ≤ 0.01; *** *P* ≤ 0.001; ns, no significance). (**B**–**E**) Survival analysis of immune checkpoint genes in the risk model. (**F**–**I**) Analysis of ESTIMATE score, immune score, stromal score and tumor purity score in the risk model (*** *P* ≤ 0.001).

**Figure 9:**
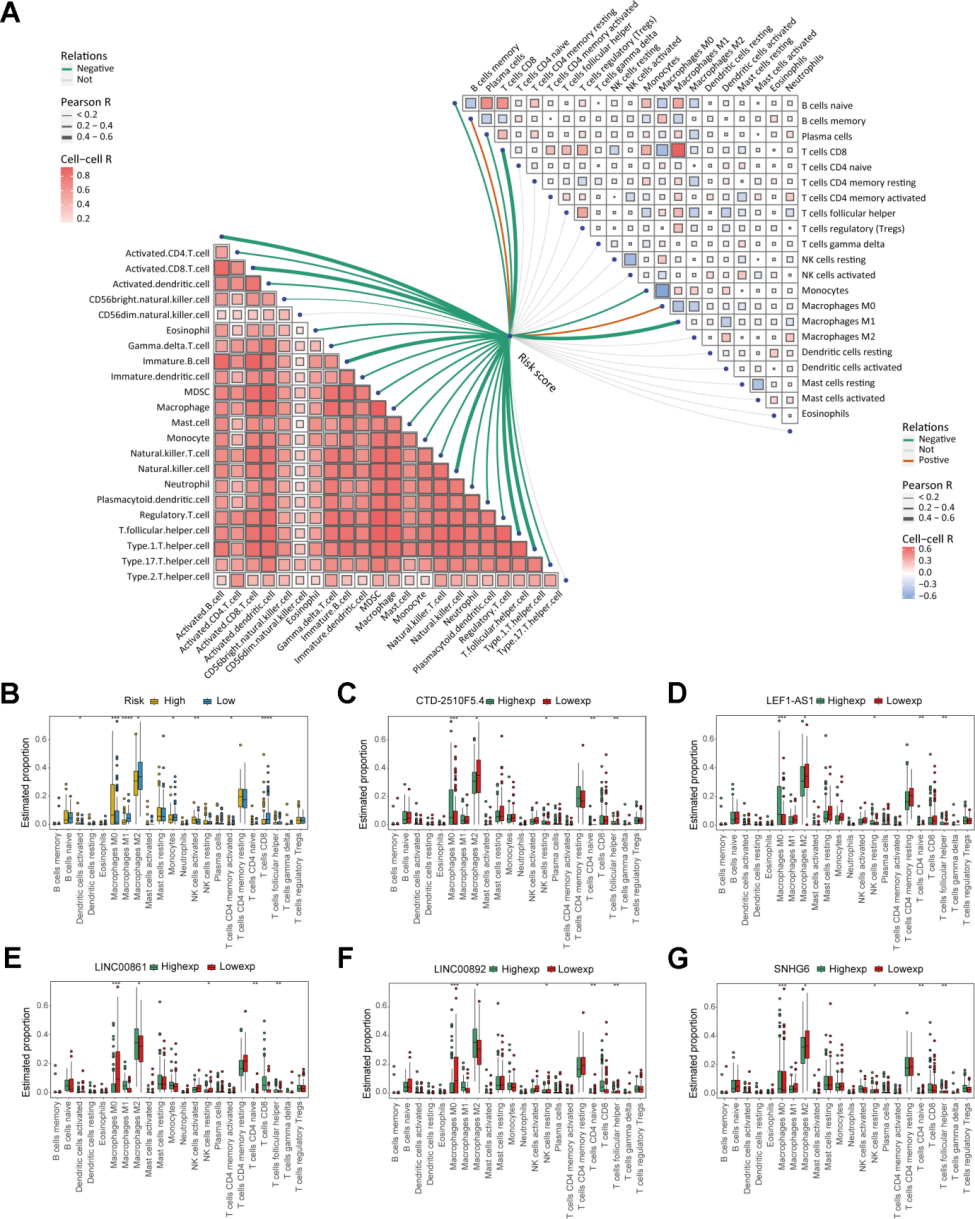
**Analysis of immune cell infiltration in the risk model.** (**A**) The correlation between risk score and immune cells. (**B**) Box plot displaying the differences in immune cell infiltration levels of risk model (* *P* ≤ 0.05; ** *P* ≤ 0.01; *** *P* ≤ 0.001; ns, no significance). (**C**–**G**) Box plot displaying the differences in immune cell infiltration levels of risk genes (* *P* ≤ 0.05, ** *P* ≤ 0.01; *** *P* ≤ 0.001; ns, no significance).

We performed GSEA on the patients of the risk model to uncover the differences in molecular functions. According to GSEA results, the low-risk group primarily relates to immune-related biological functions (Figure 7A–7D and Supplementary Table 6). Those findings suggested that immune status of tumors may serve as an important potential factor that affects prognosis of patients. Therefore, we further conducted subsequent tumor immune-related analysis. The analysis of immune checkpoints (ICPs) indicated differences in ICPs between the two risk groups of patients (Figure 8A–8E). According to the ESTIMATE algorithm, patients classified as low-risk exhibited elevated immune, stromal, and ESTIMATE scores, while demonstrating lower tumor purity scores compared to high-risk patients (Figure 8F–8I). Following this, we calculated the correlation between risk score and immune cells using the ssGSEA and CIBERSORT algorithms. The result showed a negative correlation between risk score and most immune cells (Figure 9A, 9B). This means that patients in the low-risk group have more immune cell infiltration and better immunotherapy outcomes. Additionally, we also analyzed the association between the expression level of risk genes and the immune cells. The findings indicated that the risk genes were also linked to various immune cells (Figure 9C–9G). Furthermore, we examined the drug susceptibility of the risk model (Supplementary Figure 1A–1L). The results revealed significant differences in drug susceptibility across various drugs among the two risk groups of patients. Based on the above-mentioned findings, these results demonstrate significant differences in the immune environment between the two risk groups of patients, and further provide evidence of the association between the risk model and tumor immunity.

### Verification of *in vitro* experiment about the function of key gene SNHG6 in osteosarcoma cells

**Figure 10:**
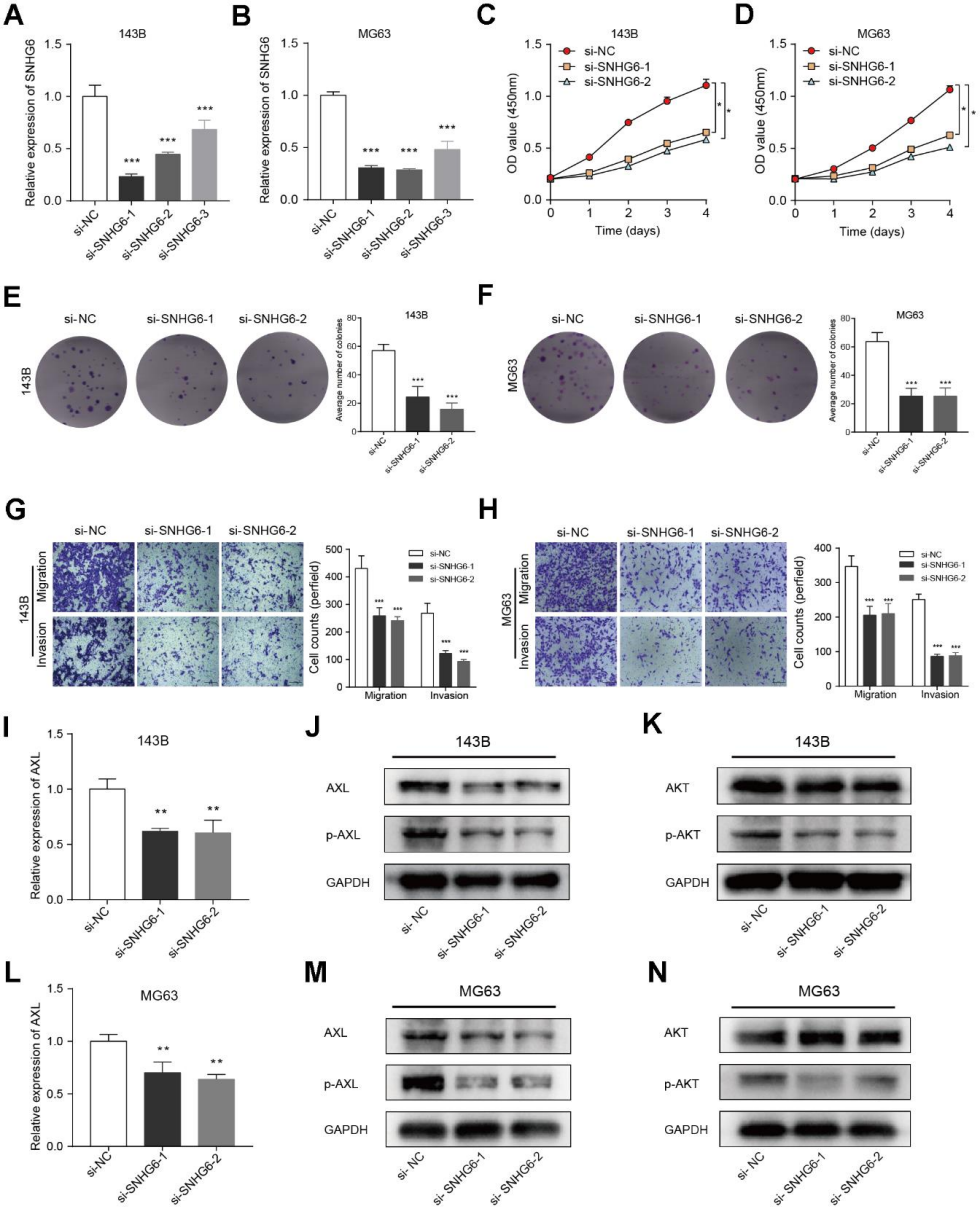
**Verification of *in vitro* experiment about the function of the key gene SNHG6 in osteosarcoma cells.** (**A**, **B**) The levels of SNHG6 mRNA in 143B and MG63 cells that were transfected with si-SNHG6 were measured using qRT-PCR (*** *P* ≤ 0.001). (**C**, **D**) The viability of 143B and MG63 cells with SNHG6 knockdown was found to be reduced according to the CCK-8 assay (* *P* ≤ 0.05). (**E**, **F**) The ability of 143B and MG63 cells to form colonies was significantly reduced after SNHG6 knockdown (*** *P* ≤ 0.001). (**G**, **H**) The migration and invasion of 143B and MG63 cells were significantly inhibited after SNHG6 knockdown (*** *P* ≤ 0.001). (**I**) The impact of SNHG6 knockdown on the AXL mRNA level in 143B cells was assessed using qRT-PCR (*** *P* ≤ 0.001). (**J**) WB analyzed the molecular weight of AXL and p-AXL in 143B cell with SNHG6 knockdown. (**K**) WB analyzed the molecular weight of AKT and p-AKT in 143B cell with SNHG6 knockdown. (**L**) The impact of SNHG6 knockdown on the AXL mRNA level in MG63 cells was assessed using qRT-PCR (*** *P* ≤ 0.001). (**M**) WB analyzed the molecular weight of AXL and p-AXL in MG63 cell with SNHG6 knockdown. (**N**) WB analyzed the molecular weight of AKT and p-AKT in MG63 cell with SNHG6 knockdown.

In order to investigate the regulation of NRlncRNAs on sarcomas, we explored the roles of NRlncRNA SNHG6 in osteosarcoma cell model. Firstly, we constructed three siRNA sequences (si-SNHG6-1, si-SNHG6-2 and si-SNHG6-3) targeting SNHG6 to attenuate the expression of SNHG6 and validated the transfection efficiency by qRT-PCR. Based on the findings, si-SNHG6-1 and si-SNHG6-2, which exhibited the most effective reduction in osteosarcoma cells 143B and MG63, were selected for further experiments (Figure 10A, 10B; *** *P* < 0.001). Next, we tested the viability of osteosarcoma cells with SNHG6 knockdown using CCK8 assay, finding that the viability was significantly decreased (Figure 10C, 10D; * *P* < 0.05). Furthermore, the colony formation assay demonstrated reduction in the colony-forming abilities of 143B and MG63 cells after SNHG6 knockdown (Figure 10E, 10F; *** *P* < 0.001). In order to further investigate the impact of SNHG6 on the migration and invasion of osteosarcoma cells, we performed transwell assay. After SNHG6 knockdown, the migration and invasion capacities of osteosarcoma cells were markedly reduced according to the results (Figure 10G, 10H; *** *P* < 0.001). These findings indicated that inhibition of SNHG6 considerably slowed the proliferation, migration, and invasion of osteosarcoma cells. The gene AXL, belonging to the Tyro3-Axl-Mer (TAM) receptor tyrosine kinase subfamily, has the ability to enhance tumor advancement and serve as an indicator of unfavorable prognosis [30]. In order to investigate the molecular regulatory mechanism of SNHG6, the connection between SNHG6 and AXL was explored using qRT-PCR and WB. Surprisingly, attenuation of SNHG6 in osteosarcoma cells reduced the expression of AXL and p-AXL, and WB results showed that the phosphorylation of AKT was also affected (Figure 10I–10N; ** *P* < 0.01). Taken together, NRlncRNA SNHG6 may be of great significance in osteosarcoma cell necroptosis through SNHG6/AXL/AKT signaling axis.

## DISCUSSION

The clinical characteristics and therapies of sarcomas vary greatly depending on different tissue origins [31]. With continuous in-depth study, the understanding of clinical, genomic, and transcriptome characteristics in several subtypes of sarcomas continues to improve, which has given some support to the diagnosis and treatment of sarcomas [32, 33]. Nevertheless, the absence of distinct genetic characteristics hinders the identification of the majority of sarcoma subtypes, leading to numerous difficulties in the precise treatment of sarcomas [32]. Recent studies have shown that immunotherapy has been proven to treat various malignant tumors, indicating that sarcomas could also benefit from the therapy targeting the immune system and TME [34, 35]. A recent study shows that the combination therapy of durvalumab and tremlimumab increased the concentration of immune cells associated with tumors in sarcomas, thus alleviating the progression of sarcomas [36]. Another clinical trial report confirmed the feasibility of combining axitinib and pembrolizumab for immunotherapy in advanced sarcoma patients [37]. Unfortunately, many patients were resistant to immunotherapies, innately or acquiredly, with one of the reasons being the lack of immune cell infiltration leading to form so-called “cold” tumors [38, 39]. Therefore, the activation or recruitment of immune cells may be a new direction for tumor immunotherapy. According to research, cell death can impact cell types and immune mechanisms in TME [10]. Necroptosis can affect tumor progression by regulating immune responses, and targeting necroptosis in tumors can profoundly affect immune cells in TME and the tumors’ response to immunotherapy [38]. Additionally, most sarcomas have a “immune cold” TME with poor response to immune checkpoint inhibitors [40]. In a word, exploration of the relationship between necroptosis markers and TME in sarcomas will help optimize subsequent immunotherapy. According to research, lncRNAs have the potential to forecast the prognosis of malignant tumors. For example, lncRNA MALAT1 was a valuable biomarker for the prognosis of osteosarcoma [41]. Ferroptosis-related lncRNA has the ability to forecast prognosis of pancreatic ductal adenocarcinoma [42]. To this end, we developed novel predictive risk model of NRlncRNAs in sarcomas and explored the value of NRlncRNAs as biomarkers in sarcomas and their role in TME.

In this study, we successfully developed a NRlncRNAs-related prognostic risk model and validated its precision through training and validation sets. Both univariate and multivariate Cox regression analyses further confirmed that the risk score was an independent prognostic factor. Furthermore, in order to show the predictive capability of the risk model regarding patients’ clinical characteristics, we developed a nomogram, which provided important clues for evaluating patients’ prognosis. The analysis of differences in clinical characteristics showed a remarkable correlation between risk score and patient’s metastatic status and OS. NRlncRNA SNHG6 revealed the highest expression in metastasis and treatment outcome. These findings indicate that the risk model we have developed has relatively stable predictive performance and universal applicability. Moreover, the risk gene SNHG6 in the model may be a potential and reliable biomarker that affects sarcoma metastasis and prognosis.

Necroptosis-related factors demonstrated significant value in predicting the prognosis, tumor immune interaction, and treatment response of sarcomas [43]. There are also studies indicating that necroptosis played an anti-tumor or pro-tumor role during the progression of sarcomas, which may be mainly related to the changes in TME caused by necroptosis [44, 45]. Thus, we analyzed the relationship between NRlncRNAs-related risk model and TME. The GSEA results indicated that many immune-related pathways were enriched in the low-risk group. This result implies the existence of potential immune response mechanisms in the risk model of NRlncRNAs. Next, ICPs differential analysis and survival analysis also suggested that our risk model provided a certain reference for sarcoma immunotherapy. Research has shown that in most sarcomas, the number of macrophages exceeded that of lymphocytes, and the number of M2 like macrophages exceeded that of M1 like macrophages [46]. Our immune cell infiltration analysis of risk model also yielded similar results. Afterwards, we assessed the susceptibility of potential anti-cancer medications in two risk groups of patients. In the low-risk group, BI-2536, Daporinad, Sepantronium bromide, UMI-77, Telomerase Inhibitor IX, and Pyridostatin exhibited superior responses according to the findings. On the other hand, the high-risk group exhibited improved responses with AZD6482, Entinostat, Mitoxantrone, Ribociclib, RVX-208, and Venetoclax. To sum up, this research showed that NRlncRNAs may impact the prognosis of sarcomas by affecting the immune microenvironment.

We screened 5 prognosis-related NRlncRNAs in the risk model, CTD-2510F5.4, LEF1-AS1, LINC00861, LINC00892, and SNHG6, among which SNHG6 exhibited the strongest correlation with patients’ OS. SNHG6 has been proven to be involved in regulating the progression of various types of tumors and to regulate multiple signaling pathways such as mTOR, PI3K/AKT, NF-κB, etc. [47]. For instance, SNHG6 was an oncogene involved in the progression of hepatocellular carcinoma, and loss of SNHG6 can inhibit liver cancer growth by inhibiting cholesterol biosynthesis [48]. SNHG6 regulates the proliferation and migration of non-small cell lung cancer [49]. SNHG6 regulated the progression of glioma through upregulation of Notch1, Sox2, and EMT [50]. Nevertheless, the function of SNHG6 as a necroptotic gene in sarcoma has not yet been elucidated. In consequence, we explored the biological behavior of SNHG6 by constructing the osteosarcoma cell model, and the results show that SNHG6 regulates the progression of osteosarcoma cells. To further explore the molecular mechanism of SNHG6, we validated the correlation between SNHG6 and AXL in osteosarcoma cells. The previous research has shown that lncRNA can regulate the progression of osteosarcoma through AXL [51]. Findings of this research indicated that the expression of AXL is directly associated with SHNG6. Therefore, SNHG6 may regulate the proliferation, migration, and invasion of osteosarcoma cells through AXL. Research has shown that AXL has the ability to facilitate the progression of osteosarcoma through the regulation of the PI3K/AKT signaling [52]. Moreover, previous reports have shown that AXL was correlated with necroptosis and inhibition of PI3K/AKT signaling can induce cell necroptosis [53, 54]. Therefore, we validated the correlation between SNHG6 and the key molecule AKT in the PI3K/AKT signaling, and the finding showed that SNHG6 regulated the expression of p-AKT. In summary, based on preliminary exploration of the molecular mechanism of NRlncRNA SNHG6, we hypothesize that SNHG6 could potentially activate the PI3K/AKT signaling through regulating AXL and may hinder the necroptosis process in osteosarcoma cells, ultimately leading to the enhancement of proliferation, migration, and invasion in osteosarcoma.

Furthermore, this study also has certain limitations. First, due to incomplete clinical information in the publicly available data of sarcomas, some clinical features cannot be analyzed. Second, although our results implied a correlation between the risk model and immune mechanisms, further experimental evidences are needed. Last, in this study, the molecular mechanism of the risk model requires more in-depth mechanism experiments to explore.

## CONCLUSIONS

This study constructed a novel NRlncRNA signature that can accurately predict sarcoma outcomes. Furthermore, we verified the accuracy of the risk model in two large cohorts and evaluated the prognosis of sarcomas by this model, as well as confirmed the biological behaviors and downstream regulatory mechanism of NRlncRNA SNHG6 in the osteosarcoma cells lines. In summary, this study provides new insights for predicting the prognosis and individualized treatment of sarcomas.

## Supplementary Material

Supplementary Figure 1

Supplementary Tables
