# Supplementary material for: Predicting prognosis and immune status in sarcomas by identifying necroptosis-related lncRNAs
Source: Aging (Albany NY). 2024 Jan 8;16(1):493–517. doi: 10.18632/aging.205383 (PMC10817413; doi:10.18632/aging.205383)
Supplement: Supplementary Figure 1 [file aging-16-205383-s001.pdf]

SUPPLEMENTARY FIGURE

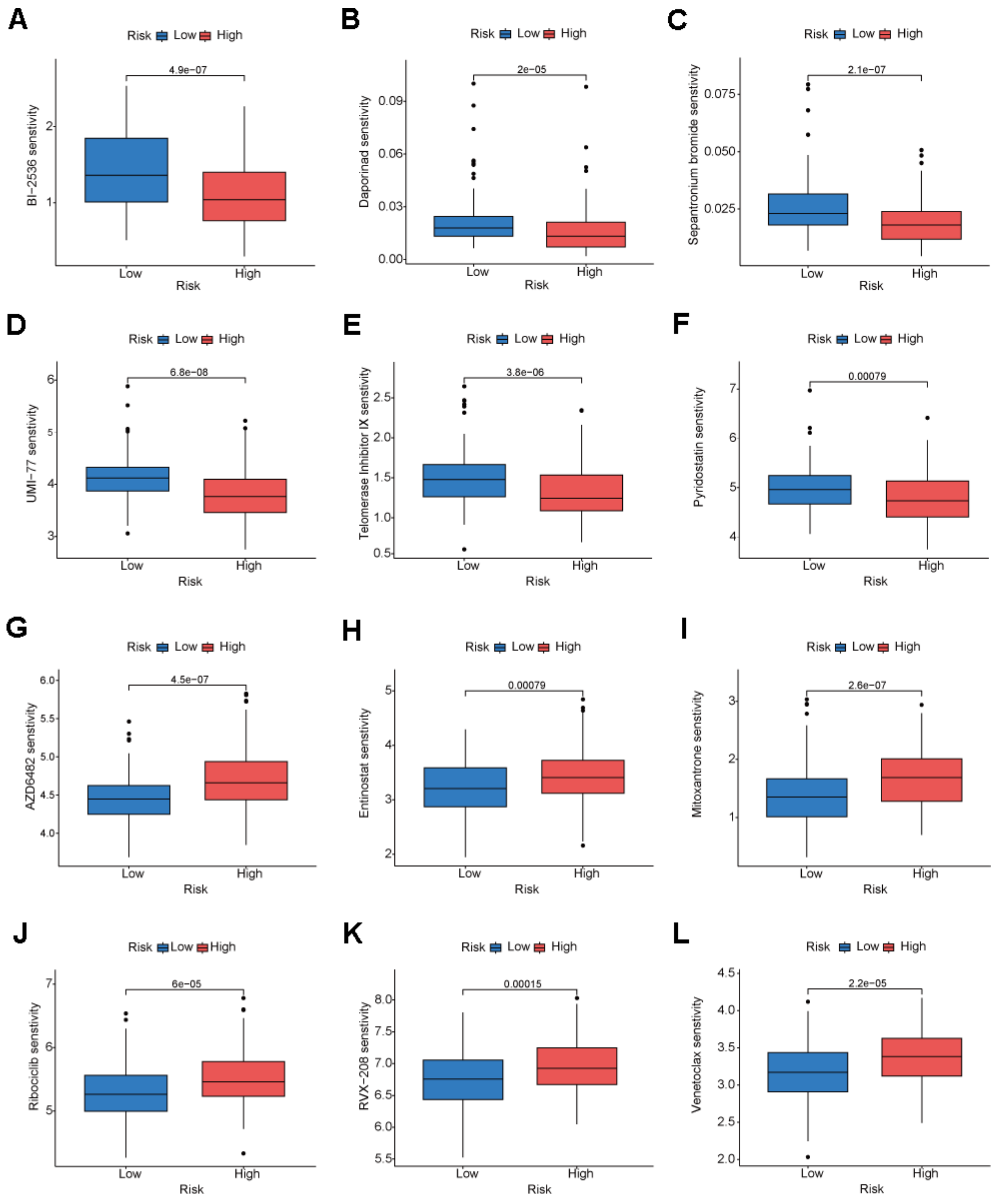

Supplementary Figure 1. (**A**–**L**) Analysis of drug susceptibility in risk model. (**A**) BI−2536, (**B**) Daporinad, (**C**) Sepantronium bromide, (**D**) UMI−77, (**E**) Telomerase Inhibitor IX, (**F**) Pyridostatin, (**G**)AZD6482, (**H**) Entinostat, (**I**) Mitoxantrone, (**J**) Ribociclib, (**K**) RVX−208, (**L**) Venetoclax.
