# Supplementary material for: Predicting prognosis and immune status in sarcomas by identifying necroptosis-related lncRNAs
Source: Aging (Albany NY). 2024 Jan 8;16(1):493–517. doi: 10.18632/aging.205383 (PMC10817413; doi:10.18632/aging.205383)
Supplement: Supplementary Tables [file aging-16-205383-s002.pdf]

SUPPLEMENTARY TABLES

Supplementary Table 1. Necroptosis-related genes.

	Gene	Total
GeneCards	RIPK1,MLKL,RIPK3,ZBP1,CASP8,TNF,CYLD,ITPK1,IPMK,MAP3K7,TRPM7,FADD,PELI1,PGLYRP1,SPATA2,CASP6,TP53,TNFRSF1A,TNFAIP3,UCHL1,SIRT3,TNIP1,STING1,SERTAD1,TRAF2,CFLAR,MAPK14,HMGB1,FAS,BIRC2,AIFM1,KLHDC10,GSK3B,XIAP,MYC,TNIP3,GJB1,DNM1L,PTGES3,BRD4,RB1,SIRT2,DAPK1,FKBP1A,MIR425,EZH2,NFE2L2,CD274,CHMP4B,CXCL5,MIR29B1,BCL2,AXL,MERTK,TYRO3,SIRT6,NAT2,SLC39A7,USP22,SFTPA1,PANX1,FLOT1,FLOT2,PDCD6IP,DIABLO,FASN,CDK9,TIMM50,SLC25A37,PPP1R3G,MIR7-1,NFKBIA,AURKC,NGFR,FMR1,GNLY,PARP1,HTRA2,HSPA5,PRKAA2,PRKAA1,PITPNA,TRAF5,METTL3,FNDC4,FNDC5,FASLG,TXN,RALBP1,TP53I3,PRKN,GSDMD,HSP90AA1,STUB1,TNFSF10,CDC37,NFKB1,RELA,TRPC6,SIRT5,MIR21,TNFRSF10B,AURKA,MYH9,CASP10,PLK1,SQSTM1,TNFRSF10A,SOAT1,HGF,SRC,ANXA1,FPR1,CASP2,MIF,KL,TNFRSF21,BCL2L1,BMI1,BBC3,AVEN,ZNF7,PTEN,ESR2,GSK3A,C5,SP1,EGR1,C7,C9,C6,ATG5,CD74,NOX4,TNFSF12,MKRN1,RCN1,UHRF1,AIFM2,SGK1,PAK1,CDC7,CERK,CTSB,CD40LG,OGT,BID,IFNB1,ID1,MIR155,TLR3,KIAA1191,TRADD,TP63,IL1A,PADI4,MIR22,MIR221,MIR214,MIR101-1,MIR485,MIR101-2,CTSD,CLEC7A,OTULIN,STAT3,HPRT1,FAP,TRIM24,CHL1,PGAM5,TGFBR1,CDKN2A,ACVR1B,PYGM,CCL2,MEFV,DPEP1,AIM2,UBR2,CHMP1A,GPX4,LAMP2,MPRIP,MTOR,BRAF,AFP,ACHE,NFAT5,VIL1,CXCL1,SHARPIN,MAPK1,CBL,GSN,TBK1,MAPK8,RARG,CTSH,CTSL,CTSS,TNFRSF25,DSTYK,RNF31,IKBKB,CHUK,BUB1B,IRAK1,MAPK3,TAB2,EIF2AK3,NQO1,HSPA8,IKBKG,BDNF,BIRC3,XBP1,BECN1,TICAM1,SOX17,HSPA4,TAB1,BNIP3,RBCK1,UBC,TAB3,AGFG1,EIF2A,TNIP2,MIB2,H1-5,DIRAS3,C20orf204,JAK1,EGFR,FLT3,IDH2,IDH1,HMOX1,MMP13,NPM1,G6PD,GJA1,TSC2,VIM,CASP1,CAD,DRD2,ATP2A1,KRT18,RPS19,TNNT2,TPM1,EPAS1,APC,AHR,ACTB,LRP1,VCP,YWHAE,YWHAG,LEF1,NOD2,PIKFYVE,EEF2,BAX,MAPKAPK2,PRDX1,KIF11,SLC16A1,KRT8,KRT5,KRT1,KRT14,NLRP3,HIF1A,HSP90AB1,HSPD1,HSPA9,PINK1,RPL5,TNFRSF1B,TPM3,TRAF3,SLC25A13,BAP1,DDX3X,RANBP2,RPL11,PRPS1,SLC25A1,MYO6,FLNC,HNRNPA2B1,HNRNPA1,IKBKE,MYH14,IL4,NUP214,PFKL,RIPK2,UBE2D3,TPM2,UGDH,TUFM,TUBB4A,TRAF6,TET2,COPB2,ARHGEF2,ATF4,EIF4EBP1,CLTC,CALM2,LTBP1,DDX5,PPP1CB,PRSS1,TUBA4A,KRT19,KRT6A,LGALS3,FUS,EMD,HSPA1A,GBE1,MYO5A,PARK7,RPS10,RPL35,S100A10,S100A4,CALM1,CCT5,AFG3L2,AHSG,MAP1B,PPM1B,PKM,PABPN1,PKP2,ULK1,XRCC6,XRCC5,UBE2L3,NDUFA4,NRIP1,NSUN2,OPTN,RPS20,STK38,SFPQ,TUBB4B,SLC25A5,LITAF,RPL15,KRT10,LGALS1,KLF6,KRT16,KRT7,MYO1C,EEF1A1,HNRNPU,FLII,ACTC1,KHDRBS1,PNKD,PSMA3,RPL7A,RPS14,RPS17,RPS24,RPS27,RPS3,RPS6,RPL13A,RPL13,TANK,TCOF1,TNFRSF8,TRIM28,TUBA1C,S100A6,TCP1,SLC30A9,CALM3,ASIC1,CADM1,CALU,RBMX,RPL22,RPL27,PCM1,PABPC1,COPA,PLEC,TUBA1B,XRN2,VDAC2,KRT2,KRT9,NCL,ELAVL1,IGF2BP3,AP2S1,PAWR,RPL26,RPL9,RPL4,RPL7,PSMC4,RPS13,RPS23,RPS26,RPS29,RPL10A,RPS12,RPL12,SVIL,SLC25A6,SNRPE,CAPZA1,EIF4B,CUL4A,CNBP,BANF1,AP2B1,MAP1LC3A,RNGTT,RPL3,PCBP1,PRPF8,PYCARD,SLC25A10,TUBB6,IGF2BP1,HNRNPF,HSPA1B,IQSEC1,IL24,MPP1,MYL6,IVNS1ABP,MVP,PDIA4,PDLIM7,RPLP2,RPS16,RPLP1,TPM4,SRSF9,SRSF1,CCNT1,DYNLL1,DNAJA1,DDX17,CCT3,BCLAF1,AGO2,RPL23A,KRT86,NME8,HNRNPH1,HNRNPL,HOOK1,HNRNPH3,FIP1L1,PDIA6,RPL6,RIOK1,RPL23,RPL28,RPL29,SSBP1,SF1,TRA2B,TRA2A,SYNCRIP,SRSF7,CCT8,ADAMTSL4,CCT6A,DNAJA2,BAG2,ATAD3A,RCC2,RFWD3,WDR77,LARP1,MYL12A,MYO1D,MYO1B,MYCBP,HNRNPM,MYL6B,GOSR1,MRPS12,RPS18,RPS8,RPS4X,RPL34,SCYL2,THRAP3,TRIP6,TMOD3,SNRPF,SRSF6,SRSF4,SRSF3,CLINT1,CEP170,AMBRA1,CPSF6,COPS2,CARD6,CAPN7,ATXN2L,ADRM1,LRRFIP2,PREB,PKP3,ZNF24,ZFP36,YBX1,ZNF217,ZC3HAV1,GCC2,FAF2,HELQ,GTF3C3,CDC42BPG,RPL17,RPS25,RPS27L,TNRC6B,TWF1,TANC2,ALYREF,CPSF2,CPSF3,DCD,MGA,RAI14,RPL38,OTUD4,PLEKHA5,TXNIP,WRNIP1,ZNF146,LIMCH1,LACTB,G3BP2,FAM83D,HEMGN,HSPBAP1,GOLGA3,HRNR,GLTP,NUDT21,SERBP1,SEC16A,SRRM2,SRP14,SRSF10,ESYT2,ERH,MLF2,RBM14,PRPF40A,POF1B,VBP1,YTHDC1,AKAP8L,AKNA,TRAFD1,CALML5,PNN,ELP1,KCTD5,RBM25,UBL4A,APOOL,CRTAM,CTAG2,LRRC59,PPP1R12C,PALMD,TLE6,ZSCAN20,ZKSCAN4,H1-2,RPL39,SP6,CHTOP,UBAP2,ZAN,GSDME,TMEM44,CEP44,ATP5F1C,LZTS3,RHOXF2,TMEM263,ZNF391,H1-10,UTP11,H2BC12,H2AC12,OBI1,MAIP1,RBM14-RBM4,MIR137,MIR148A	601
MSigDB	FADD,FAS,FASLG,MLKL,RIPK1,RIPK3,TLR3,TNF	8

GO	SLC25A4,BIRC2,BIRC3,BOK,CASP8,CAV1,CYLD,FZD9,YBX3,RIPK1,FADD,CFLAR,ARHGEF2,SPATA2,RBCK1,RIPK3,PELI1,ZBP1,MIR101-1,MIR101-2,MIR103A1,MIR103A2,MIR107,MIR214,MIR22,MIR221,MIR485,FAS,FASLG,CD14,ITPK1,PYGL,TLR3,TLR4,TNF,TP53,DNM1L,PPIF,LY96,TRPM7,TICAM1,PGAM5,MLKL,IPMK,TICAM2	45
KEGG	TNF,TNFRSF1A,TRADD,TRAF2,TRAF5,RIPK1,BIRC2,BIRC3,XIAP,RBCK1,RNF31,SHARPIN,SPATA2L,SPATA2,CYLD,FADD,CASP8,CFLAR,RIPK3,CYBB,CAMK2A,CAMK2D,CAMK2B,CAMK2G,SLC25A4,SLC25A5,SLC25A6,SLC25A31,PPID,VDAC1,VDAC2,VDAC3,GLUD2,GLUD1,GLUL,PYGL,PYGM,PYGB,MAPK8,MAPK10,MAPK9,FTH1,FTL,PLA2G4E,PLA2G4A,JMJD7-PLA2G4B,PLA2G4B,PLA2G4C,PLA2G4D,PLA2G4F,ALOX15,CAPN1,CAPN2,SMPD1,MLKL,PGAM5,DNM1L,NLRP3,PYCARD,CASP1,IL1B,CHMP2A,CHMP2B,CHMP3,RNF103-CHMP3,CHMP4B,CHMP4A,CHMP4C,CHMP6,VPS4B,VPS4A,CHMP1B,CHMP1A,CHMP5,CHMP7,TRPM7,IL1A,IL33,HMGB1,TNFSF10,TNFRSF10A,TNFRSF10B,FASLG,FAS,FAF1,IFNA1,IFNA2,IFNA4,IFNA5,IFNA6,IFNA7,IFNA8,IFNA10,IFNA13,IFNA14,IFNA16,IFNA17,IFNA21,IFNB1,IFNG,IFNAR1,IFNAR2,IFNGR1,IFNGR2,JAK1,JAK2,JAK3,TYK2,STAT1,STAT2,STAT3,STAT4,STAT5A,STAT5B,STAT6,IRF9,EIF2AK2,TLR4,TICAM2,TICAM1,TLR3,ZBP1,USP21,SQSTM1,HSP90AA1,HSP90AB1,TNFAIP3,PARP1,BID,BAX,AIFM1,H2AX,H2AC20,H2AC12,H2AC1,H2AW,H2AB3,H2AC8,H2AC4,MACROH2A2,MACROH2A1,H2AC19,H2AJ,H2AB1,H2AC17,H2AC18,H2AC11,H2AC21,H2AZ2,H2AC7,H2AZ1,H2AC15,H2AC6,H2AC13,H2AC14,H2AC16,H2AB2,PPIA,BCL2	159

Supplementary Table 2. Sequences of primers and siRNA.

	Names	Sequences
Sequences of primers (5'-3')	SNHG6(Forward)	ACGCGGCATGTATTGAGCATATAGG
	SNHG6(Reverse)	TGCCACACTTGAGGTAACGAAGC
	AXL(Forward)	TGTGCTGTCAGACGATGGGATG
	AXL(Reverse)	CGGATGCTTGCGAGGTGAGG
	GAPDH(Forward)	ACACCCACTCCTCCACCTTTG
	GAPDH(Reverse)	TCCACCACCCTGTTGCTGTAG
Target sequences of siRNA (5'-3')	si-SNHG6-1	ACGCGGCATGTATTGAGGTTGCTGT
	si-SNHG6-2	TGAGGTGAAGGTGTATGAAAGTCAT
	si-SNHG6-3	TCACGCGGCATGTATTGAGCATATA

Supplementary Table 3. GO and KEGG enrichment of necroptosis-related mRNAs.

	ID	Description	p-value
Top 5 results for GO enrichment of necroptosis-related mRNAs	GO:0042110	T cell activation	0.000
	GO:0007159	leukocyte cell-cell adhesion	0.000
	GO:0032944	regulation of mononuclear cell proliferation	0.000
	GO:0050863	regulation of T cell activation	0.000
	GO:0070663	regulation of leukocyte proliferation	0.000
	GO:0009897	external side of plasma membrane	0.000
	GO:0000775	chromosome, centromeric region	0.000
	GO:0030667	secretory granule membrane	0.000
	GO:0042613	MHC class II protein complex	0.000
	GO:0000776	kinetochore	0.000
	GO:0140375	immune receptor activity	0.000
	GO:0023023	MHC protein complex binding	0.000
	GO:0004896	cytokine receptor activity	0.000
	GO:0019955	cytokine binding	0.000
	GO:0019864	IgG binding	0.000
Top 20 results for KEGG enrichment of necroptosis-related mRNAs	hsa04060	Cytokine-cytokine receptor interaction	0.000
	hsa04640	Hematopoietic cell lineage	0.000
	hsa05152	Tuberculosis	0.000
	hsa04650	Natural killer cell mediated cytotoxicity	0.000
	hsa04514	Cell adhesion molecules	0.000
	hsa05150	Staphylococcus aureus infection	0.000
	hsa04145	Phagosome	0.000
	hsa04380	Osteoclast differentiation	0.000
	hsa05140	Leishmaniasis	0.000
	hsa04658	Th1 and Th2 cell differentiation	0.000
	hsa04659	Th17 cell differentiation	0.000
	hsa05323	Rheumatoid arthritis	0.000
	hsa04660	T cell receptor signaling pathway	0.000
	hsa05416	Viral myocarditis	0.000
	hsa05330	Allograft rejection	0.000
	hsa05320	Autoimmune thyroid disease	0.000
	hsa05321	Inflammatory bowel disease	0.000
	hsa05340	Primary immunodeficiency	0.000
	hsa05332	Graft-versus-host disease	0.000
	hsa04940	Type I diabetes mellitus	0.000

Supplementary Table 4. Cox regression analyses on various clinical characteristics and risk scores.

	Univariate analysis				Multivariate analysis			
Characteristic	p-value	HR	lower .95	upper .95	p-value	HR	lower .95	upper .95
RiskScore	0	7.966	3.345	18.97	0	18.755	6.435	54.668
Age	0.004	1.023	1.007	1.04	0.856	0.96	0.62	1.486
Gender	0.438	0.853	0.571	1.275	0.001	1.029	1.012	1.047
Lipomatous Neoplasms	0.125	1.718	0.861	3.427	0.022	2.298	1.129	4.677
Myomatous Neoplasms	0.258	1.451	0.762	2.763	0.111	1.707	0.884	3.296
Nerve Sheath Tumors	0.535	1.494	0.421	5.3	0.346	1.857	0.513	6.726
Soft Tissue Tumors and Sarcomas (NOS)	0.063	2.131	0.96	4.729	0.228	1.635	0.735	3.633
Synovial-like Neoplasms	0.565	1.395	0.449	4.33	0.877	0.906	0.261	3.15

Supplementary Table 5. Baseline data chart of clinical characteristics.

	Alive (n=158)	Dead (n=98)	Total (n=256)
**Gender**			
Female	82(51.9%)	57(58.2%)	139(54.3%)
Male	76(48.1%)	41(41.8%)	117(45.7%)
**Age**			
Mean	58.4	63.5	60.4
Median	60	66	60.5
Min	20	29	20
Max	87	89	89
<40	18(11.4%)	4(4.1%)	22(8.6%)
40-60	59(37.3%)	35(35.7%)	94(36.7%)
60-80	71(44.9%)	46(46.9%)	117(45.7%)
≥80	10(6.3%)	13(13.3%)	23(9.0%)
**Histologic Subtype**			
Conventional	42(26.6%)	22(22.4%)	64(25%)
Poorly differentiated	16(10.1%)	18(18.4%)	34(13.3%)
Well differentiated	3(1.9%)	1(1.0%)	4(1.6%)
Not reported	97(61.4%)	57(58.2%)	154(60.2%)
**Race**			
Asian	4(2.5%)	1(1.0%)	5(2.0%)
Black or African American	11(7.0%)	7(7.1%)	18(7.0%)
White	139(88.0%)	85(86.7%)	224(87.5%)
Not reported	4(2.5%)	5(5.1%)	9(3.5%)
**Metastatic**			
YES	19(17.3%)	37(57.8%)	56(32.2%)
NO	91(82.7%)	27(42.2%)	118(67.8%)
**Recurrence**			
YES	8(7.5%)	21(33.3%)	29(17.1%)
NO	99(92.5%)	42(66.7%)	141(82.9%)
**Disease Type**			
Fibromatous Neoplasms	28(17.7%)	12(12.2%)	40(15.6%)
Lipomatous Neoplasms	34(21.5%)	25(25.5%)	59(23.0%)
Myomatous Neoplasms	62(39.2%)	41(41.8%)	103(40.2%)
Nerve Sheath Tumors	6(3.8%)	3(3.1%)	9(3.5%)
Soft Tissue Tumors and Sarcomas, NOS	22(13.9%)	13(13.3%)	35(13.7%)
Synovial-like Neoplasms	6(3.8%)	4(4.1%)	10(3.9%)
**Treatment Outcome**			
Complete Response	119(78.8%)	6(13.6%)	125(64.1%)
Partial Response	0(0.0%)	2(4.5%)	2(1.0%)
Progressive Disease	25(16.6%)	33(75.0%)	58(29.7%)
Stable Disease	7(4.6%)	3(6.8%)	10(5.1%)

Supplementary Table 6. GSEA results of risk model.

		ID	Enrichment Score	p-value
Top 10 GSEA results for KEGG	High risk group	KEGG_ALLOGRAFT_REJECTION	-0.812	0
		KEGG_ANTIGEN_PROCESSING_AND_PRESENTATION	-0.751	0
		KEGG_CHEMOKINE_SIGNALING_PATHWAY	-0.562	0
		KEGG_CYTOKINE_CYTOKINE_RECEPTOR_INTERACTION	-0.627	0
		KEGG_GRAFT_VERSUS_HOST_DISEASE	-0.826	0
		KEGG_HEMATOPOIETIC_CELL_LINEAGE	-0.662	0
		KEGG_INTESTINAL_IMMUNE_NETWORK_FOR_IGA_PRODUCTION	-0.787	0
		KEGG_LEISHMANIA_INFECTION	-0.732	0
		KEGG_NATURAL_KILLER_CELL_MEDIATED_CYTOTOXICITY	-0.624	0
		KEGG_TOLL_LIKE_RECEPTOR_SIGNALING_PATHWAY	-0.688	0
	Low risk group	KEGG_ALLOGRAFT_REJECTION	0.812	0
		KEGG_ANTIGEN_PROCESSING_AND_PRESENTATION	0.751	0
		KEGG_CHEMOKINE_SIGNALING_PATHWAY	0.562	0
		KEGG_CYTOKINE_CYTOKINE_RECEPTOR_INTERACTION	0.627	0
		KEGG_GRAFT_VERSUS_HOST_DISEASE	0.826	0
		KEGG_HEMATOPOIETIC_CELL_LINEAGE	0.662	0
		KEGG_INTESTINAL_IMMUNE_NETWORK_FOR_IGA_PRODUCTION	0.787	0
		KEGG_LEISHMANIA_INFECTION	0.732	0
		KEGG_NATURAL_KILLER_CELL_MEDIATED_CYTOTOXICITY	0.624	0
		KEGG_TOLL_LIKE_RECEPTOR_SIGNALING_PATHWAY	0.688	0
Top 10 GSEA results for GO	High risk group	GOBP_ACTIVATION_OF_IMMUNE_RESPONSE	-0.781	0
		GOBP_IMMUNE_RESPONSE_REGULATING_CELL_SURFACE_RECEPTOR_SIGNALING_PATHWAY	-0.789	0
		GOBP_IMMUNOGLOBULIN_PRODUCTION	-0.867	0
		GOBP_LYMPHOCYTE_MEDIATED_IMMUNITY	-0.779	0
		GOBP_PRODUCTION_OF_MOLECULAR_MEDIATOR_OF_IMMUNE_RESPONSE	-0.793	0
		GOCC_IMMUNOGLOBULIN_COMPLEX	-0.949	0
		GOCC_IMMUNOGLOBULIN_COMPLEX_CIRCULATING	-0.942	0
		GOCC_T_CELL_RECEPTOR_COMPLEX	-0.931	0
		GOMF_ANTIGEN_BINDING	-0.901	0
		GOMF_IMMUNOGLOBULIN_RECEPTOR_BINDING	-0.941	0
	Low risk group	GOBP_B_CELL_RECEPTOR_SIGNALING_PATHWAY	0.888	0
		GOBP_HUMORAL_IMMUNE_RESPONSE_MEDIATED_BY_CIRCULATING_IMMUNOGLOBULIN	0.863	0
		GOBP_IMMUNOGLOBULIN_PRODUCTION	0.867	0
		GOBP_LYMPHOCYTE_MEDIATED_IMMUNITY	0.779	0
		GOBP_PHAGOCYTOSIS_RECOGNITION	0.876	0
		GOCC_IMMUNOGLOBULIN_COMPLEX	0.949	0
		GOCC_IMMUNOGLOBULIN_COMPLEX_CIRCULATING	0.942	0
		GOCC_T_CELL_RECEPTOR_COMPLEX	0.931	0
		GOMF_ANTIGEN_BINDING	0.901	0
		GOMF_IMMUNOGLOBULIN_RECEPTOR_BINDING	0.941	0
